# Systematic Review of Nutrient Profile Models Developed for Nutrition-Related Policies and Regulations Aimed at Noncommunicable Disease Prevention —An Update

**DOI:** 10.1016/j.advnut.2023.08.013

**Published:** 2023-08-31

**Authors:** Caroline Martin, Mylène Turcotte, Juliette Cauchon, Amélie Lachance, Sonia Pomerleau, Véronique Provencher, Marie-Ève Labonté

**Affiliations:** 1 Centre Nutrition, santé et société (NUTRISS), Institute of Nutrition and Functional Foods (INAF), Université Laval, Québec, QC, Canada; 2 School of Nutrition, Université Laval, Québec, QC, Canada

**Keywords:** food quality, food regulation, healthfulness, healthy food, nutrient profiling, nutritional quality, nutrition policy, public health, systematic review, validation

## Abstract

Nutrient profiling (NP) models are useful for characterizing the healthfulness of foods and for underpinning various nutrition-related public health strategies. Recently, there has been a rapid increase in the number of NP models developed by different organizations worldwide. A systematic review (SR) summarizing the key characteristics of NP models with applications in government-led nutrition policies was carried out in 2016 and published by Labonté et al. [4]. Given the continuous proliferation of NP models, the current study aimed to update this SR. Systematic searches were performed in databases of both the peer-reviewed (*n =* 7) and grey (*n =* 1) literature to identify publications related to NP published between May 2016 and September 2020. The full text of relevant publications was assessed independently by 2 reviewers to build a list of potential models. Each model was classified as “already identified in the original SR” or as “newly identified.” The eligibility of the “newly identified” models, and of some models excluded from the previous SR because their details were not known at that time, were then assessed independently by 2 reviewers based on pre-established criteria. A total of 151 potential NP models were assessed for eligibility, of which 93 were “newly identified,” 28 were originally excluded from the previous SR, and 30 were identified from additional online searches during the eligibility assessment stage. Twenty-six models met the inclusion criteria. Their most frequent applications were food labeling (*n =* 17) and regulation of food marketing to children (*n =* 7). They all included nutrients to limit, with sodium, saturated fat, and total sugars being the most frequently considered. Content or face validity testing was conducted for 11 (42%) of the included models. As NP models are increasingly used worldwide to support public health strategies, having an up-to-date resource listing them and detailing their characteristics is crucial. PROSPERO #CRD42021259041.

**Statement of Significance**

The current update of a previous systematic review of NP models identified 26 new models over a 4-y period, primarily representing nutrient-specific rating systems built for front-of-pack food labeling and restricting food marketing to children. These new models have mostly been found in regions not identified in the previous review and built based on other existing models, therefore reinforcing the importance of having performed this update.

## Introduction

Nutrient profiling (NP) algorithms aim to characterize the overall nutritional quality (healthfulness) of foods and beverages [1]. They are generally based on a food product’s content in multiple nutrients, some of which may be to encourage (e.g., fiber, protein) and others to limit (e.g., sugars, sodium). The increasing inclusion of food components (e.g., additives, percent composition in plant-derived ingredients such as fruits, vegetables, nuts, and legumes) in NP algorithms suggests they should now be referred to as “food classification systems,” although the current paper adheres to the more conventional term of NP models even in cases where algorithms feature more than just nutrients. NP is primarily relevant to the field of public health nutrition when there is a need to define as clearly and objectively as possible what represents a “less” or “more” nutritious food in the context of various nutrition-related policies and regulations [2]. NP models are therefore built to be used, among several other possible applications, in the regulation of food marketing to children, front-of-package (FOP) food labeling or nutrition claims, in food taxation or subsidies, in food assistance programs, as part of procurement standards in public institutions, and even in product reformulation [3, 4].

In 2018, our group published a systematic review that described and summarized the key characteristics of NP models developed or endorsed by governmental or intergovernmental organizations as part of nutrition-related policies and regulations [4]. The focus was on government-based NP models, given that the aim was to create an up-to-date resource particularly intended for policymakers to assist them in the selection of models appropriate for their needs in regard to nutrition-related policies and regulations. In this context, it was assumed that nonauthoritative based models would be less likely to be adopted and used by government bodies, explaining why they were not retained. Out of 387 potential NP models identified, 78 were included in the review. It was found that nearly three-quarters (73%) of the included models had been introduced in the previous decade, namely between 2006 and mid-2016. Our group had, therefore, highlighted that nutrient profiling is a rapidly evolving field in which brand new NP models or NP models adapted from existing ones might be proposed for use at any moment. In support of this statement, Kanter et al. [5] have reported that the number of FOP food labeling systems has proliferated in recent years: 12 new systems have been introduced worldwide only between 2016 and mid-2019. Of note, Canada has also recently proposed a “FOP nutrition symbol” meant to appear on packaged foods when they exceed predefined cut-offs for one or more of 3 nutrients to limit, namely total sugars, saturated fat, and sodium [6]. It is additionally worth pointing out that information was lacking about some NP models in the SR by Labonté et al. [4], particularly those only available in a “draft” or “proposed” version. One cannot rule out the possibility that these models might have officially been adopted for use since then, with detailed information on such models now available. With these considerations in mind, it became imperative to update the original review by Labonté et al. [4]. The current systematic review, therefore, aims to summarize and discuss key characteristics of NP models with applications in government-led nutrition policies and regulations that have been published in their draft or final versions since mid-2016.

## Methods

The current systematic review was carried out in accordance with the Preferred Reporting Items for Systematic Reviews and Meta-Analyses (PRISMA) statement [7]. A pre-established protocol has been registered in PROSPERO (2021: CRD42021259041) [8].

**FIGURE 1:**
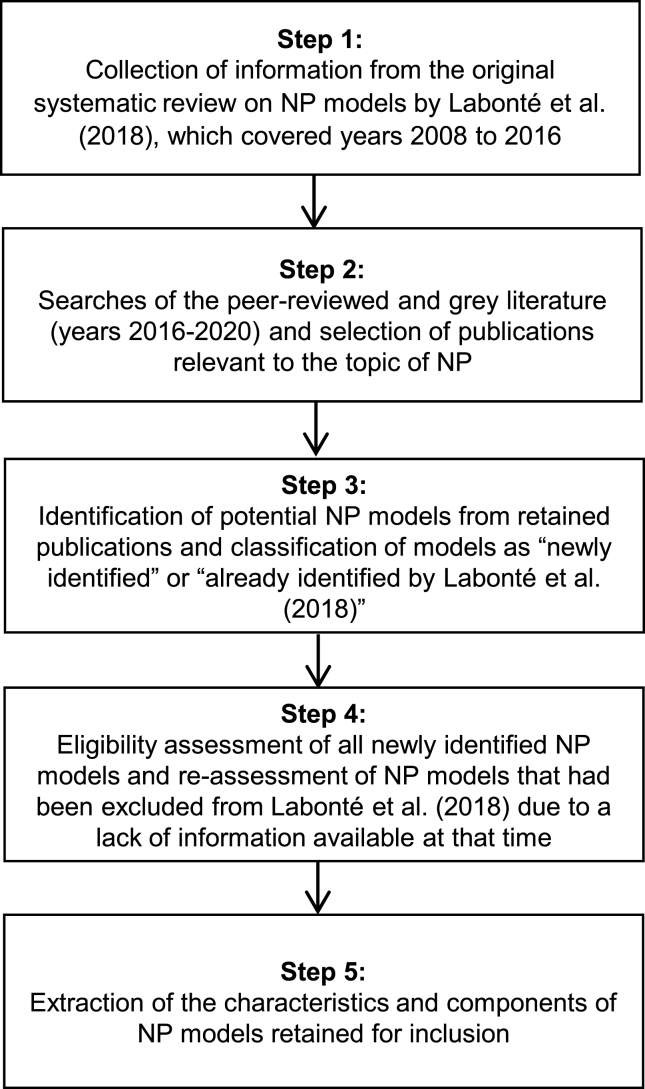
Steps of the systematic review. Abbreviations used: NP, nutrient profile.

As mentioned earlier, the current systematic review builds on and updates a previous systematic review on NP models by Labonté et al. [4]. As in Labonté et al. [4], the primary objective of the current work was to identify NP models existing worldwide for application in various types of government-led nutrition-related policies and regulations aimed at health promotion and noncommunicable disease (NCD) prevention. The methodology used (e.g., eligibility criteria for publications and NP models) was, therefore, similar to the one of the original review [4]. The current update more specifically followed 5 main steps, which are listed in Figure 1 and further detailed below.

### Step 1: Collection of information from the original systematic review by Labonté et al. (2018)

In the original review by Labonté et al. [4], 309 out of 387 potential NP models had been excluded. As part of the current work, the review team aimed to retrieve, within these 309 excluded models, all models that had specifically been discarded due to criterion G (i.e., details of the model are not known) as defined in Table 1, either alone or in combination with criterion H or J. These models had been excluded by Labonté et al. [4] at the time of the original review, but there was a possibility that detailed information on those models had become available since then. The aim of retrieving these models was, therefore, to re-assess their eligibility as part of Step 4 described below.

**TABLE 1:** Criteria used for the eligibility assessment of all potential NP models identified^1^

Reason	Inclusion criteria	Exclusion criteria
A	Models allowing for the classification or categorization of individual foods.	Models only allowing for the classification or categorization of combinations of foods (i.e., meals or diets, such as the Healthy Eating Index).
B	Models integrating data from more than one nutrient or food component to produce a single overall score or categorization, or models with separate sets of criteria for multiple nutrients or food components (e.g., Traffic Light System in which the levels of each of the nutrients considered are interpreted separately).	Models in which only a single nutrient or food component is used, as focusing on only one aspect of the nutritional composition can mask the overall nutritional quality of a food product (e.g., nutrient content claim; reformulation targets for single nutrients such as sodium; Whole Grain Stamp).
C	Models with a food focus that also use criteria based on nutrients and other food components.	Models with a food focus that do not use criteria based on the amounts of nutrients and other food components (e.g., a model which only states that soft soda cannot be advertised to children without considering the underlying nutritional composition of the products).
D	Models in which the output is a score or classification and includes at least a modest interpretative element.	Models in which the output shows little or no interpretative element (e.g., models only repeating the amounts of some nutrients found in the Nutrition Facts Table, or models showing a percentage of GDAs, a percentage of DVs or the GDAs/DVs themselves).
E	Models developed or endorsed^2^ by governmental or intergovernmental organizations and having applications in government-led nutrition policy and regulation, including, but not limited to: - Food-certification schemes/front-of-pack labeling - Standards for food advertising or marketing - Regulation of health and nutrition claims - Food procurement regulations/food quality standards for public institutions (e.g., schools, workplaces, hospitals, armed services, prisons, elderly care homes) - Food taxation - Food subsidies - Welfare support schemes - Food fortification - Nutritional surveillance - Reformulation	Models developed by different types of organizations (e.g., commercial; nongovernmental; academia, etc.) that are not endorsed^2^ by government bodies (e.g., models developed by the food industry for their own voluntary marketing restrictions; models developed by heart foundations for food-certification schemes)
F	Models intended for national or international use, or for use in a jurisdiction with responsibility for the relevant food policy or regulation (e.g., models developed by states or provinces responsible for school food standards).	Models intended for use at a very specific / narrow level (e.g., municipal).
G	Details of the model are publicly available in the peer-reviewed or grey literature (e.g., government documents/websites, theses, etc.).	Details of the model are not known because they are not publicly or freely available, or they could not be found, therefore not allowing for the appropriate use or adaptation of a model or appropriate evaluation of its construct and components.
H	Final versions of models which are currently in use or draft models that have been proposed for use within the last 3 to 5 y.	Discontinued models no longer in use, or proposed models that were never implemented.
I	Models that do not duplicate information included previously.	Models duplicating information from another model (e.g., an exact same model is described in multiple documents, but under slightly different names).
J	Full details of the model are available in English or French. Full details of the model are available on a website that offers automatic translation.	Full details available in another language than specified in the left column. The website (or PDF link) on which full details of the model are available does not offer an option for automatic translation.
K	N/A	“Not relevant”: This represents the situation where it is found, during eligibility assessment, that a policy, regulation, standard, scheme, etc., initially considered as a potential nutrient profile model actually does not correspond to such a model (i.e., does not use any criteria to classify foods, either food-based or nutrient-based). For example, this could be a Code in which it is found, when reviewing the source document, that there is a total ban of the commercial advertising of any type of product to children, food or not. Therefore, this means that no nutrient profile model is used as part of this Code to determine which foods can or cannot be advertised to children.

1 Letters are used to indicate the reason(s) for exclusion in the list of excluded models (Supplemental Table 3). Abbreviations used: DV, Daily Value; GDA, Guideline Daily Amount; N/A, not applicable; NP, nutrient profile.

2 In the systematic review by Labonté et al. [4] and in the current review, "endorsed" refers to models that are used by governmental or intergovernmental organizations or that are made reference to in government publications in relation to ≥ 1 of the above applications, but that were not specifically developed by such organizations. For example, a government body may have mandated another organization for the development of a model aimed at supporting one of their nutrition-related policies.

### Step 2: Searches of the peer-reviewed and grey literature and selection of publications relevant to the topic of NP

#### Literature searches

Searches have been carried out in various electronic databases to identify publications relevant to the topic of NP in both the scientific and grey literature: a) Peer-reviewed literature: PubMed, MEDLINE, EMBASE, COCHRANE, CINHAL, PSYC-INFO, Google Scholar; and b) Grey literature: Web of Science. The following broad search terms, which were essentially the same as in the original review by Labonté et al. [4], have been used in each database: nutrient profil∗ OR nutritional profil∗ OR nutrition profile∗. An example of the search strategy specific to each database is provided as supplementary data (Supplemental Tables 1 and 2).

Of note, the literature searches were conducted on 2 different occasions. A first set of searches was conducted on December 2, 2018, and a second one on September 2, 2020. The first set of searches was limited to papers published between May 26, 2016, and December 2, 2018. The start date represented the moment at which the last searches had been conducted in the previous systematic review by Labonté et al. [4]. The second set of searches covered the period between the end date of the first set (December 2, 2018) and September 2, 2020, and results from both sets of searches were then combined. All searches were performed by one author (CM).

No restriction on language was imposed during the searches. All search results were exported to citation management software [EndNote X6; Clarivate Analytics].

#### Publication Selection

One of the authors (CM) first removed duplicates within the combined search results using the function “Find duplicate” in the citation management software. A manual verification was further performed to ensure the removal of any remaining duplicates.

In terms of eligibility criteria for publications, similar to Labonté et al. [4], there was no restriction on the type of publication to be included in the systematic review. For example, research articles of any study design, government documents, reports, theses, etc., were all deemed eligible as long as their screening revealed that they comprised terms related to NP or NP applications in their title, abstract, summary, or table of content (i.e., at least one term between nutrient, nutritional, nutrition, or food AND at least one term between profil∗, criter∗, scor∗, standard∗, requirement∗, program∗, guideline∗, schem∗, healthy, healthi∗, healthful∗, classification, advertis∗, market∗, labeling/labeling, subsid∗, tax∗, govern∗). Such publications have been retained for further evaluation based on their full text. Publications have been discarded only when it was clear that they were not relevant to the topic of the review. Reasons for excluding a publication comprised, for example, publication about the nutrient profile of animal food or diets; studies in the field of agriculture, such as comparisons between the nutrient profile of different varieties of plants or seeds; tools that assess the quality of food exclusively according to criteria other than nutritional value (e.g., NOVA food processing system [9]).

To enhance objectivity and avoid mistakes, the screening of the identified publications was performed independently by 2 review team members (CM + SP or CM + AL). In case of a disagreement, a third review team member (MT or MEL) was involved.

### Step 3: Identification of potential NP models from retained publications and classification of models as “newly identified” or “already identified by Labonté et al. (2018)”

#### Identification of potential NP models

This step consisted of evaluating the full text of all publications retained after the screening stage (step 2). It has allowed to confirm the eligibility of the publications and, therefore, to identify all potential NP models that were mentioned, described, tested, or used to answer a specific research question in a given publication, in any part of it (i.e., title, abstract, introduction, methods, results, discussion or conclusion). As in Labonté et al. [4], any classification or scoring system, standard, requirement, program, guideline, regulation, legislation, etc. that potentially included the use of nutritional criteria to evaluate the nutritional quality of food products was recorded as a potential NP model. This step was performed independently by 2 review team members (CM + AL), and a third review team member (MT) was consulted in cases of disagreement. Then, 2 team members (CM + AL or CM + MT) independently captured the names of all potential NP models included in each publication to build a list of potential models. They also evaluated the presence of possible duplicates in the model names, with help from the references provided for each model, and decided on a single name for each potential model.

#### Classification of NP models as “already identified in the systematic review by Labonté et al. (2018)” or as “newly identified”

All potential NP models identified as part of the full-text assessment stage were then classified independently by 2 review team members (CM + MT) either as “already identified in the systematic review by Labonté et al. [4]” or as “newly identified.” Disagreements were resolved by consensus, and if consensus was not reached, a third review team member (MEL) was involved. For all models that were not previously identified in Labonté et al. [4], or if it was not possible at this point to establish whether a model corresponded either to a previously identified model or to a newly identified model, a new identifier (i.e., model number) was given to the model in prevision of eligibility assessment. Models have been numbered starting from no. 396, which represented the number next to the last identifier given by Labonté et al. [4].

### Step 4: Eligibility assessment of all newly identified NP models and reassessment of models that had been excluded from Labonté et al. (2018) due to a lack of information available at that time

#### NP model eligibility criteria

As in Labonté et al. [4], the current update includes publicly available NP models developed or endorsed by government bodies. These models had to be built for application in nutrition-related policies or regulations at the provincial/state level or higher and had to provide an interpretation of the nutritional quality of individual food products based on multiple (i.e., ≥2) nutrients or food components, either in the form of a summary indicator system (e.g., global assessment illustrated as a “healthy” logo) or in the form of a nutrient-specific system (e.g., a traffic light system for multiple nutrients assessed individually). The included NP models had to represent the final version in use or a draft version proposed for use within the last 3–5 y, with details available in English or in French. Eligibility criteria for the NP models are fully described in Table 1.

#### Eligibility assessment

All newly identified models and each model that had been excluded from Labonté et al. [4] specifically due to criterion G, either alone or in combination with criterion H or J (as described under Step 1), were assessed according to the eligibility criteria in Table 1. Eligibility assessment was conducted with the use of information from the publications retrieved in the literature searches, supplemented, if necessary, with information obtained from online searches about specific models (e.g., searches on Google or government websites) or from requests sent to an author or a contact person in organizations that developed the models. These contact persons could direct us to the appropriate location of a publicly available document about a given NP model.

Eligibility assessment was completed independently by 2 team members (CM + MT or CM + JC). Evaluations were then compared, and discordances were resolved by consensus or by involving a third review team member (MEL). Models that met all inclusion criteria as of November 29, 2021, were included in the review and retained for data extraction. Models that were not eligible based on ≥ 1 of the exclusion criteria were kept in a list of excluded models (Supplemental Table 3). This list comprises the model number, the model’s name, the source reference(s), the date of last access, reason(s) for exclusion, details on reason(s) for exclusion, and additional information on the model (if relevant).

#### Additional potential NP models identified during the eligibility assessment stage

Through additional documentation reviewed (e.g., searches on Google and Google Scholar, on governmental websites, and on PubMed), the above eligibility assessment stage allowed the identification of additional potential NP models that had not been retrieved as part of the literature searches. These additional potential NP models were therefore assessed independently by 2 team members (CM + JC) against the eligibility criteria defined in Table 1.

### Step 5 – Data extraction

Data on all included NP models were extracted into a Microsoft Excel Worksheet using the same fields as in Labonté et al. [4]. Data extraction fields included, for example, the model number; model name; type and name of the organization(s) that developed the model; possible applications (purposes) of the model; a list of food categories included; list of nutrients to limit and nutrients to encourage; reference amounts; outputs; and information on validation. Data extraction of all models was performed independently by 2 review team members (JC + CM or JC + MT). Disagreements were resolved by consensus or by involving a third review team member (MEL).

In the current article, extracted data have been separated into different tables to facilitate data synthesis, reading, and understandability. The article does not present all of the extracted data. However, a searchable Excel database including all possible fields can be made available upon request to the corresponding author.

## Results

### Literature search results and publication selection

**FIGURE 2:**
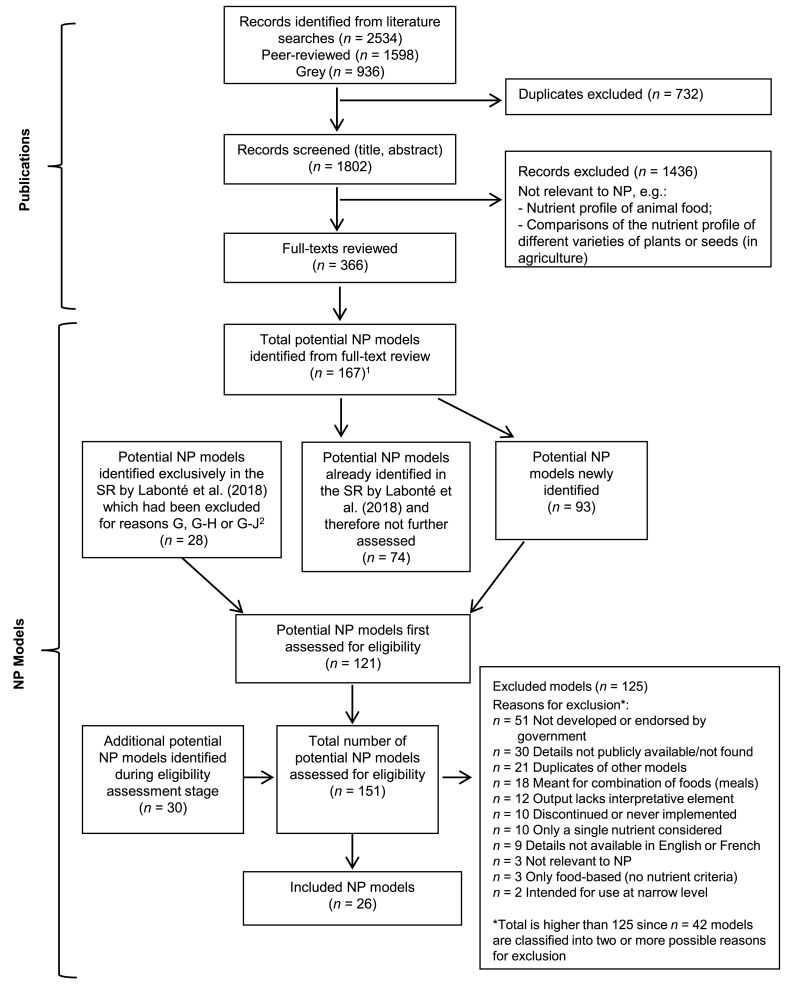
Flow diagram of the publications and NP models selection. Data are current as of November 29, 2021. ^1^ Of note, the number of full texts reviewed is not related to the number of potential NP models identified from these publications. ^2^ These 28 models correspond to models that had been excluded from Labonté et al. (2018) specifically due to criterion G, either alone or in combination with criterion H or J (see Table 1), because information on these models was not publicly available at that time. It was decided to re-assess the eligibility of those models as part of the current systematic review, since information about them might have become available. Abbreviations used: NP, nutrient profile; SR, systematic review.

Literature searches allowed identifying a total of 2534 publications, from which 1598 records originated from the peer-reviewed literature and 936 records originated from the grey literature (Figure 2). Following the removal of duplicates, 1802 records remained for the screening stage based on titles and abstracts or summaries, which led to 366 publications being retained for further evaluation based on their full text.

### Identification of NP models and eligibility assessment

The full-text assessment of selected publications allowed the identification of 167 potential NP models, of which 93 represented newly identified potential models. The other 74 models had already been identified in the previous systematic review by Labonté et al. [4] and, therefore, did not need to be assessed for eligibility (Figure 2). Of note, models that had already been identified in the original review but comprised algorithms that have been updated since then (e.g., Health Star Rating system, no. 196) were not considered here as new potential models, since their main characteristics, such as their name, country of origin, and primary application remained the same. In addition to the 93 newly identified models, 28 models found exclusively in Labonté et al. [4] which had been excluded because their details were not available at that time (i.e., models with reasons of exclusion G, G-H or G-J, see Step 1 and Table 1 for more details) were considered as potential NP models in the current review. This led to 121 potential NP models. However, 30 additional potential NP models were further identified during the eligibility assessment stage of these 121 models (see Step 4). Therefore, eligibility assessment finally occurred for a total of 151 models, and 26 of these met the inclusion criteria. Of the 125 models that have been excluded, 41% (*n* = 51) were discarded because they were not developed or endorsed by a governmental organization (Figure 2). Thirty-four percent (34%) of the excluded models had more than one reason for exclusion (*n* = 42). Reasons for the exclusion of each model are provided as supplementary data (Supplemental Table 3).

### The main characteristics of included NP models

#### Possible applications of NP models

**FIGURE 3:**
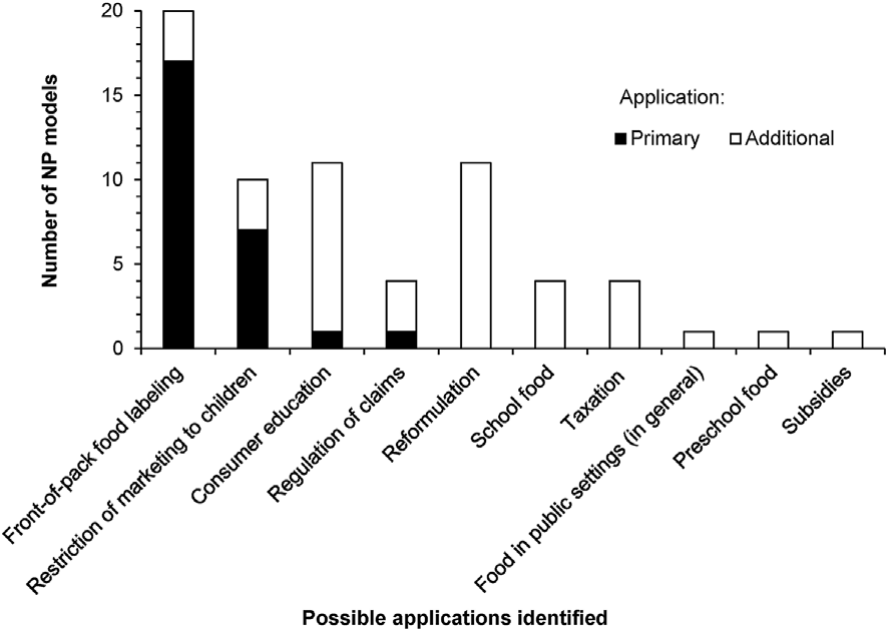
Number of nutrient profile models associated with each possible application identified. An application is defined as a purpose for which a nutrient profile model was built. Applications are sorted first by descending order of the number of models per primary application (black bars), and second by descending order of the number of models per additional application (white bars). Each model is associated with only one primary (i.e. main) application; the addition of black bars therefore equals 26. However, a model could also be associated to one or more additional (i.e. secondary) application. The number of additional applications per model ranged between 0 and 7, explaining why the total of additional applications is not equal to the total number of models. Further details on the possible applications of the models and specific model numbers associated with each application are provided in Table 2.

A primary application, defined as the main nutrition-related policy or regulation for which a model was built, was identified for each included NP model. As in Labonté et al. [4], primary applications were determined using information extracted from each model based on their source references. Given that NP models may sometimes be built for use in more than one context, other applications were also identified for some of the included models. Figure 3 shows the primary and additional applications, whenever relevant, of the 26 included models. The most common primary application was FOP food labeling, present in almost two-thirds of the NP models (*n* = 17, 65%), followed by the restriction of food and beverage marketing to children (*n* = 7, 27%), regulation of health or nutrition claims (*n* = 1, 4%), and consumer education (*n* = 1, 4%). Seventeen of the included models (65%) had at least one additional possible application, the most common additional applications being reformulation (*n* = 11, 42% of all included models) and consumer education (*n* = 10, 38%)*.* Details on the specific model numbers associated with each possible application are provided in Table 2.

**TABLE 2:** Applications listed for the 26 included NP models and model numbers associated with each application^1^

Applications^2^	Primary application, *n*	Model number(s)	Additional application, other than primary, *n*	Model number(s)	Total: primary + additional application, *n*
Food labeling:					
Food-certification scheme / front-of-pack labeling	17	213, 221, 414, 445, 483, 484, 485, 496, 499, 501, 502, 503, 504, 506, 511, 512, 515	3	296, 477, 516	20
Regulation of claims (e.g., health and/or nutrient claims)	1	513	3	501, 506, 516	4
Food in public settings:					
Schools	0		4	336, 477, 485, 516	4
Preschools	0		1	485	1
In general	0		1	516	1
Restriction of the promotion/marketing of foods to children	7	296, 333, 336, 415, 477, 481, 516	3	213, 445, 485	10
Consumer education	1	451	10	221, 296, 483, 496, 499, 501, 502, 506, 515, 516	11
Taxation	0		4	296, 336, 477, 516	4
Reformulation	0		11	221, 296, 414, 451, 477, 485, 496, 499, 502, 515, 516	11
Subsidies	0		1	485	1

1 An application represents the purpose for which a nutrient profile model was built. Each model is associated with only one primary application; therefore the sum of the number of models per primary application equals 26. An additional application represents one that is specified in the source reference of a model in addition to its primary application (e.g. model no. 515 is primarily meant for front-of-pack food labeling, but also has reformulation and consumer education as additional applications). For a given model, the number of additional applications could range between none and 7.

2 In the previous systematic review by Labonté et al. [4], a higher number of possible applications had been identified (e.g. Food in public settings: recreational facilities (e.g., national parks), health facilities (e.g., hospitals), government facilities (e.g., food procurement; food sold in cafeterias), vending machines (in various settings); Food system/surveillance). Since none of the models included in the current review had these applications as a primary or additional application, they were not listed in the current table. However, a new application, consisting of Food in public settings (in general), has emerged and has been added to this table.

#### Characteristics related to the development of NP models

Table 3 describes the various characteristics related to the development of included NP models, including each model’s specific name. Models are first listed according to their primary application (decreasing order) and then by their country of origin (alphabetical order). Half of the included models originated from 2 regions: Asia (*n* = 7, 27%) followed by the Americas (*n* = 6, 23%). The Slovenia Traffic Light System (no. 451) was the only model entirely built by nongovernmental or academic organizations and, therefore, considered as endorsed by the Slovenian government. Indeed, this government cofinanced the model’s development because they believed it would lead to beneficial changes in consumer food choices and encourage the food industry to reformulate their products. All other models (*n* = 25, 96%) were developed at least in part by a governmental or intergovernmental organization. Fifteen models (58%) were based on at least one other existing model identified in the current review and/or by Labonté et al. [4].

**TABLE 3:** Characteristics of the development of each included NP model (*n* = 26)^1^

Country (state/province if applicable)	Model number	Model name (reference(s))	Organization type	Organization name	Year of introduction or seminal publication	Model derived from other model(s)? [yes or no]^2^	Model that served in the development of other model(s)? [yes or no]^3^
Front-of-pack food labeling (*n* = 17)							
Brazil	506	Brazil Warning Labels ([10], [11], [12])	Govt or intergovt	The Brazilian Health Regulatory Agency^4^	2020	No	No
Brunei Darussalam	501	Brunei Healthier Choice Logo (13, 14)	Govt or intergovt	Brunei's Ministry of Health	2017	73	No
Canada	414	Canada Proposed Warning Labels (15)	Govt or intergovt	Health Canada	2018^5^	No	No
Croatia	496	Croatia Healthy Living Mark (16, 17)	Govt or intergovt	Croatian Institute of Public Health	2016	No	No
India	213	India Proposed Nutrient Profile Model (18)	Govt or intergovt	India's Ministry of Health and Family Welfare^6^	2019	No	No
Iran	503	Iran Traffic Light Labelling System ([19], [20], [21])	Govt or intergovt	Iran's Ministry of Health and Medical Education	2014	41	No
Israel	485	Israel Green Label (22)	Govt or intergovt; Academic	Israel Ministry of Health (Food Control Services and Nutrition Division), as part of a Scientific Committee also including leading nutrition and medical professionals from academia and the healthcare system	2020	No	No
Israel	221	Israel Red Warning Labels ([23], [24], [25])	Govt or intergovt	Israel Ministry of Health	2017	156	No
Kingdom of Saudi Arabia	511	Saudi Arabia Proposed Traffic Light Labelling System (26)	Govt or intergovt	Saudi Food and Drug Authority (SFDA)	2018	41	No
Malaysia	502	Malaysia Healthier Choice Logo ([27], [28], [29])	Govt or intergovt	Malaysia's Ministry of Health - Nutrition Division	2017	53, 73, 334 (therefore indirectly 335, itself based on 62, 251, 5 and 11)	No
Mexico	445	Mexican Warning Labels (Nutrisello) (30)	Govt or intergovt	Mexican Ministry of Health	2020	No	No
Peru	483	Peru Warning Labels (31)	Govt or intergovt	Peru's Ministry of Health	2017	156, 388	No
Sri Lanka	504	Sri Lanka Traffic Light Labelling System ([32], [33], [34])	Govt or intergovt	Sri Lanka Ministry of Health, Nutrition and Indigenous Medicine with the Food Advisory Committee	2019	No	No
Thailand	515	Thailand Healthier Choice Logo (35, 36)	Govt or intergovt; Academic	Thailand's Ministry of Public Health, Thai Health Promotion Foundation, The Institute of Nutrition^7^, and Thailand's Food Service FDA	2016	No	No
Tunisia	512	Tunisia Health Logo ([37], [38], [39], [40])	Govt or intergovt	Tunisia's Ministry of Health	2015	69, 333	No
Uruguay	484	Uruguay Warning Labels (41)	Govt or intergovt	Uruguay Ministry of Public Health	2017	156	No
Zambia	499	Zambia Good Food Logo ([42], [43], [44])	Govt or intergovt; Commercial	Zambia's Ministry of Health^8^, Scaling Up Nutrition Business Network (SBN), Zambia Bureau of Standards	2020	53	No
Restriction of marketing to children (*n* = 7)							
Canada	415	Canada Proposed Nutrient Profile Model for Marketing to Children ([45], [46], [47])	Govt or intergovt	Health Canada	2016	5, 335 (itself based on 62, 251, 5 and 11), 388	No
China (Taiwan)	481	Taiwan Recommendations on Sugar, Fat, Saturated fatty acids (48)	Govt or intergovt	Taiwan Ministry of Health and Welfare	2016	No	No
International	333	WHO Nutrient Profile Model for the Eastern Mediterranean Regional Office (WHO-EMRO) (38, 49)	Govt or intergovt	WHO Regional Office for the Eastern Mediterranean Region in collaboration with the Department of Nutrition for Health and Development at WHO headquarters	2014 (field tested); 2017 (published)	335 (itself based on 62, 251, 5 and 11)	512
International	336	WHO Nutrient Profile Model for the South-East Asia Regional Office (WHO-SEARO) (50)	Govt or intergovt	WHO Regional Office for the South-East-Asia Region (SEARO)	2016 (field tested); 2017 (published)	334 (itself based on 335), 335 (itself based on 62, 251, 5 and 11)	296
International	516	WHO Nutrient Profile Model for the African Region (51)	Govt or intergovt	WHO Regional Office for Africa in collaboration with Member States and the Department of Nutrition for Health and Development at WHO headquarters	2019	336 (itself based on 334 and 335)	No
Samoa	477	Samoa Nutrient Profile System for Identifying Healthier and Less Healthy Food Options (52)	Govt or intergovt; Academic	Melbourne's Global Obesity Centre, Samoa Ministry of Health, Australia's Centre for Pacific Island Studies, Netherlands' School of Nutrition and Translational Research in Metabolism/School for Public Health, Primary Care and Menzies Centre for Health Policy	2018	No	No
Sri Lanka	296	Sri Lanka Nutrient Profile Model (53)	Govt or intergovt	Sri Lanka Ministry of Health, Nutrition, and Indigenous Medicine, in collaboration with WHO	2018	336 (itself based on 334 and 335)	No
Regulations of claims (e.g., health and/or nutrients) (*n* = 1)							
Tunisia	513	Tunisia Nutrient Content Claims (54)	Govt or intergovt	Tunisia's Minister of Trade and Crafts, Public Health, Industry, Energy and Small and Medium Enterprises	2008	No	No
Consumer education (*n* = 1)							
Slovenia	451	Slovenia Traffic Light System ([55], [56], [57])	NGOs (non-profit research institute and non-profit consumer education foundation); Academic (University-affiliated research institute)^9^	The Jožef Stefan Institute, the Slovenian Consumers' Association (ZPS), and the Institute of Nutrition	2019	41	No

1 Abbreviations used: Govt, Governmental; FDA, Food and Drug Administration; intergovt, intergovernmental; NGO, nongovernmental organization.

2 Model derived from one or more other models identified as part of the systematic review by Labonté et al. [4] or the current review; [yes (indicated by model numbers) or no].

3 Model that served at least in part as the basis for the development of ≥1 other included model; [yes (indicated by model numbers) or no].

4 Agência Nacional de Vigilância Sanitária - ANVISA.

5 Introduction in 2018 with pre-consultation white paper published in 2016.

6 Food safety and standards authority of India.

7 Institute of Nutrition at the Mahidol University.

8 Zambia National Food and Nutrition Commission.

9 The Ministry of Health of the Republic of Slovenia has cofinanced the project. It appears that they have supported this model’s development because they believed it would lead to beneficial changes in consumer food choices and encourage the food industry to reformulate their products.

#### Characteristics related to the components of NP models

Table 4 summarizes the main components of all included NP models overall and according to each of the 4 primary applications identified, whereas Table 5 presents the main characteristics of each model separately. Additional information on the specific nutrients and food components considered in each model and on the specific types of reference amounts or units considered (e.g., per 100 g, per 100 ml, per serving) is provided in Supplemental Table 4 and Supplemental Table 5, respectively.

**TABLE 4:** Main characteristics of included NP models, overall and according to the 4 primary applications identified^1^

Characteristics	Total (n = 26)	Front-of-pack food labeling (n = 17)	Restriction of marketing to children (n = 7)	Regulation of claims (n = 1)	Consumer education (n = 1)
Type of rating system					
Summary rating of the nutritional quality	13 (50%)	6 (35%)	7 (100%)	—	—
Nutrient-specific rating	11 (42%)	9 (53%)	—	1 (100%)	1 (100%)
Combination of both	2 (8%)	2 (12%)	—	—	—
Type of output					
Classification	25 (96%)	16 (94%)	7 (100%)	1 (100%)	1 (100%)
Score	—	—	—	—	—
Combination of both	1 (4%)	1 (6%)	—	—	—
Food categories^2^					
Level at which nutrient criteria are applied					
Major only	13 (50%)	8 (47%)	3 (43%)	1 (100%)	1 (100%)
Major and sub	6 (23%)	2 (12%)	4 (57%)	—	—
Major, sub, and sub-sub	3 (12%)	3 (18%)	—	—	—
Sub only	2 (8%)	2 (12%)	—	—	—
Sub and sub-sub	2 (8%)	2 (12%)	—	—	—
Total number of food categories, including nutrient criteria (min-max)	1-84	1-84	1-29	2	2
Nutrients and food components					
Number/type of nutrients and food components may vary across the model’s food categories/types of food products evaluated	15 (58%)	9 (53%)	5 (71%)	0 (0%)	1 (100%)
Inclusion of nutrients^3^ to limit	26 (100%)	17 (100%)	7 (100%)	1 (100%)	1 (100%)
Number of nutrients^3^ considered (min-max)	3 - 10	3 - 10	4 - 8	6	4
Top 3 nutrients^3^ considered (% of models that include the nutrient)^4^	1. Total sodium (100%)	1. Total sodium (100%)	1. Saturated fat (100%)	N/A	N/A
	2. Saturated fat (88%)	2. Total sugars (88%)	2. Total sodium (100%)		
	3. Total sugars (88%)	3. Saturated fat (82%)	3. Total fat (86%)		
			4. Total sugars (86%)		
Inclusion of nutrients^3^ to encourage	8 (31%)	6 (35%)	0 (0%)	1 (100%)	1 (100%)
Number of nutrients^3^ to encourage (min-max)	1 - 16	3 - 16	N/A	2	1
Top 3 nutrients^3^ considered (% of models that include the nutrient)^4^	1. Fiber (88%)	1. Fiber (100%)	N/A	N/A	N/A
	2. Calcium (50%)	2. Calcium (67%)			
	3. Iron (50%)	3. Iron (67%)			
	4. Protein (50%)				
Reference amounts/units					
Top 3 types of reference amounts or other units considered (% of models that include the reference amount/unit)^5^	1. Per 100 g (88%)	1. Per 100 g (88%)	1. Per 100 g (86%)	N/A	N/A
	2. Per 100 ml (65%)	2. Per 100 ml (65%)	2. Per 100 ml (57%)		
	3. Per 100 kcal or % of energy (31%)	3. Presence or absence of a nutrient or a food component (35%)	3. Per 100 kcal or % of energy (29%)		
	4. Presence or absence of a nutrient or a food component (31%)		4. Per other prespecified amount (g or ml) (29%)		
			5. Per serving (29%)		
			6. Presence or absence of a nutrient or a food component (29%)		
Validity testing and other characteristics					
Some degree of validity testing identified (e.g., content, construct/convergent, face, and/or criterion-related/predictive validity)	11 (42%)	5 (29%)	6 (86%)	0 (0%)	0 (0%)
Number of models derived from other models, either included or excluded, identified as part of this review or the review by Labonté et al. [4]	15 (58%)	9 (53%)	5 (71%)	0 (0%)	1 (100%)
Number of models that served at least in part as the basis for the development of ≥1 other included model	2 (8%)	0 (0%)	2 (29%)	0 (0%)	0 (0%)

1 Values are n (%) of models unless stated otherwise. Table 5 presents a more detailed summary of the key characteristics of each model. Additional details on the specific nutrients and food components included in each model and on the specific types of reference amounts and other evaluation units used in each model can be found in Supplemental Table 4 and Supplemental Table 5. Abbreviations used: max, maximum; min, minimum; N/A, not applicable.

2 Major food categories represented the first and sometimes only level of categories described in a model. In some models, ≥ 1 major category was subdivided into subcategories. In a few cases, ≥ 1 subcategory was further subdivided into sub-subcategories (e.g., Zambia Good Food Logo, model no. 499, in which the major category of fruits and vegetables includes 4 subcategories and one of these also includes 2 sub-subcategories).

3 Also implies food components.

4 Nutrients are listed in descending order of the proportion of models that include them. Alphabetical order was used when proportions were equal; therefore, the number of nutrients listed may be > 3. N/A was used when the number of models evaluated was equal to 1.

5 Reference amounts and other evaluation units are listed in descending order of the proportion of models that include them. Alphabetical order was used when proportions were equal; therefore, the number of reference amounts/units listed may be > 3. N/A was used when the number of models evaluated was equal to 1.

**TABLE 5:** Summary characteristics of the 26 included NP models^1^

Model number	Model name	Type of model^2^	Output^3^	Category level(s) at which nutrient criteria are applied^4^	Number of food categories with nutrient criteria^5^	Model including only nutrients/food components to limit (A) or nutrients/food components to limit and to encourage (B)^6^	Number/type of nutrients and food components may vary across the model's food categories/type of food product evaluated (Y/N)	Total nutrients/ food components to limit^7^	Total nutrients/ food components to encourage^7^	Type of reference amount/unit considered			
										Per 100 g and/or per 100 ml	Per 419 kJ (i.e., 100 kcal) or % of energy	Per serving	Other reference amount or unit^8^
Front-of-pack food labeling (*n* = 17)													
506	Brazil Warning Labels	B	A	Major	2	A	N	3	0	Y	—	—	—
501	Brunei Healthier Choice Logo	C	A	Sub & sub-sub	84	B	Y	9	3	Y	—	Y	Y
414	Canada Proposed Warning Labels	B	A	Major	3	A	N	3	0	—	—	Y	Y
496	Croatia Healthy Living Mark	A	A	Sub & sub-sub	50	B	Y	10	4	Y	—	—	Y
213	India Proposed Nutrient Profile Model	C	A	Major, sub & sub-sub	34	A	Y	4	0	Y	—	—	—
503	Iran Traffic Light Labelling System	B	A	Major	2	A	N	4	0	Y	—	Y	—
485	Israel Green Label	A	A	Major & sub	23	A	Y	5	0	Y	—	—	—
221	Israel Red Warning Labels	B	A	Major	2	A	N	3	0	Y	—	—	—
511	Saudi Arabia Proposed Traffic Light Labelling System	B	A	Major	2	A	N	4	0	Y	—	—	—
502	Malaysia Healthier Choice Logo	A	A	Sub	47	B	Y	4	16	Y	—	—	Y
445	Mexican Warning Labels (Nutrisello)	B	A	Sub	6 - 3^9^	A	Y	7	0	Y	Y	—	Y
483	Peru Warning Labels	B	A	Major	2	A	N	4	0	Y	—	—	—
504	Sri Lanka Traffic Light Labelling System	B	A	Major	2	A	N	3	0	Y	—	—	—
515	Thailand Healthier Choice Logo	A	C	Major, sub & sub-sub	27	B	Y	7	4	Y	Y	Y	Y
512	Tunisia Health Logo	A	A	Major & sub	22 10	B	Y	9	5	Y	Y	Y	Y
484	Uruguay Warning Labels	B	A	Major	1	A	N	4	0	Y	Y	—	—
499	Zambia Good Food Logo	A	A	Major, sub & sub-sub	40	B	Y	6	7	Y	Y	—	Y
Restriction of marketing to children (*n* = 7)													
415	Canada Proposed Nutrient Profile Model for Marketing to Children	A	A	Major	2	A	N	4	0	Y	Y	Y	Y
481	Taiwan Recommendations on Sugar, Fat, Saturated fatty acids	A	A	Major	1	A	N	4	0	—	Y	Y	—
333	WHO Nutrient Profile Model for the Eastern Mediterranean Regional Office (WHO-EMRO)	A	A	Major & sub	22	A	Y	8	0	Y	—	—	—
336	WHO Nutrient Profile Model for the South-East Asia Regional Office (WHO-SEARO)	A	A	Major & sub	25	A	Y	8	0	Y	—	—	Y
516	WHO Nutrient Profile Model for the African Region	A	A	Major & sub	25	A	Y	8	0	Y	—	—	Y
477	Samoa Nutrient Profile System for Identifying Healthier and Less Healthy Food Options	A	A	Major	5	A	Y	4	0	Y	—	—	—
296	Sri Lanka Nutrient Profile Model	A	A	Major & Sub	29	A	Y	8	0	Y	—	—	Y
Regulations of claims (e.g., health and/or nutrients) (*n* = 1)													
513	Tunisia Nutrient Content Claims	B	A	Major	2	B	N	6	2	Y	Y	—	—
Consumer education (*n* = 1)													
451	Slovenia Traffic Light System	B	A	Major	2	B	Y	4	1	Y	—	—	—

1 Abbreviations used: N, No; NP, Nutrient Profile; Sub, subcategory; sub-sub, sub-subcategory; Y, Yes.

2 A, summary indicator system; B, nutrient-specific system; C, a combination of both.

3 A, classification; B, score; C, a combination of both.

4 Major, sub-, or sub-subcategory level. Major food categories represented the first and sometimes only level of categories described in a model. In some models, a major category was subdivided into subcategories. In a few cases, a subcategory was further subdivided into sub-subcategories (e.g., Zambia Good Food Logo, no. 499, in which the major category of Source of Carbohydrates includes 8 subcategories and one of these also includes 3 sub-subcategories). It should be noted that certain models included “exempted” and/or “excluded” foods within their list of food categories, whereas other models did not and therefore presented “exempted” and/or “excluded” foods separately.

5 It should be noted that certain models established nutrient criteria at the major, subcategory, and/or sub-subcategory levels. As such, the number of food categories at any level may not correspond to the number of food categories with nutrient criteria. Although the term “nutrient” criteria is used, the data presented here may include nutrient-based and/or food-based criteria because certain models provided a combination of nutrient-based and/or food-based criteria for all or a selection of food categories.

6 None of the included models considered only nutrients and food components to encourage.

7 Total excludes optional nutrients and food components that can be considered as part of some models but that are not mandatory. Details on the specific nutrients and food components considered in each model are provided in Supplemental Table 4.

8 Details on the other possible reference amounts/units for each model are provided in Supplemental Table 5. These may include, e.g., the evaluation of some nutrients or food components in terms of other prespecified amounts (in g or mL; e.g., per 250 mL), % of total fat, % daily value or reference daily intake, % weight/quantity of a component in a product (e.g., % fruit content) or the presence (or absence) of a nutrient or food component in a product (e.g., no added sweeteners).

9 The number of categories depends on the regulation's different phases of implementation. Phases 1 and 2 include 6 categories, while Phase 3 includes 3 categories.

10 Model no. 69: 1 category; Model no. 333: 22 categories.

Summary rating systems based on the amounts of multiple nutrients or food components represented 50% of the models (*n* = 13), whereas 11 models (42%) provided a nutrient-specific rating of the nutritional quality of food products. Only 2 models, built for FOP food labeling, represented a combination of these 2 types of rating systems by generating both a summary rating and a nutrient-specific evaluation of food products (Table 4). The output of 25 models (96%) consisted of a classification (e.g., Croatia Healthy Living Mark, no. 496, which classifies food products as eligible or not to carry a healthy logo). For the only remaining model (i.e., Thailand Healthier Choice Logo, no. 515), a combination of both types of output was used and varied depending on the food category evaluated. The output represented only a classification for some food categories, whereas it took the form of a score that was then translated into a classification for some other categories (TABLE 4, TABLE 5).

There was a large variation between the different NP models in the number and type of food categories that included nutrient criteria. Thirteen models (50%) included only major food categories, of which the number ranged between 1 and 5 (e.g., 2 categories: Processed Solid Food and Beverages, in the Peru Warning Labels, no. 483). The other half of the NP models included at least one subcategory in addition to major categories (e.g., Israel Green Label, no. 485, in which the major category of Dairy products is divided into a) Liquid milk, b) Fermented milk products, and c) Cheeses). Five of these models (19%) also included sub-subcategories (e.g., Zambia Good Food Logo, no. 499, in which the major category of fruits and vegetables includes 4 subcategories and one of these subcategories also includes 2 sub-subcategories). Within a single model, nutrient criteria could be applied at any food category level (i.e., major, sub-, or sub-subcategory level). For example, the Sri Lanka Nutrient Profile Model, no. 296, applies nutrient criteria at both the major and subcategory levels. For instance, the major category of Confectionary applies the same set of criteria to any type of confectionary, whereas the major category of Ready-to-eat savories does not include nutrient criteria *per se*. It is rather subdivided into the subcategories of a) Potato, cereal, or starch-based products, and b) Processed nuts, which each include different nutrient criteria. As a result, across all models, the total number of categories that included different nutrient criteria within a specific model ranged between 1 and 84. The largest ranges (maximum–minimum) in the number of food categories with nutrient criteria were specifically observed in NP models built for FOP food labeling (Table 4).

All models included nutrients or food components to limit (*n =* 26, 100%), whereas 8 models (31%) also included nutrients or food components to encourage (Table 4, Supplemental Table 4). The number of nutrients or food components to limit varied between 3 and 10 across the different models, and the top 3 consisted of sodium (100% of models), saturated fat (88%), and total sugars (88%). Among the 8 models that included nutrients or food components to encourage, the number of such nutrients ranged from 1 to 16, and the top consisted of fiber (88%), calcium (50%), iron (50%), and protein (50%). The overall number and type of nutrients and food components considered in a model varied across the model’s food categories for 58% of the models (*n* = 15; e.g., India Proposed Nutrient Profile Model, no. 213, in which saturated fat and sodium are considered only in some specific categories, whereas *trans* fat and added sugars are considered in all categories).

The main types of reference amounts (or other units) taken into account in the NP models were, in descending order, per 100 g (*n* = 23; 88%), per 100 ml (*n* = 17; 65%), per 419 kJ (100 kcal) or % of energy (*n* = 8; 31%), and the presence (or absence) of a given nutrient or food component in a food product (e.g., no added sweeteners; *n* = 8; 31%). Three models used a single type of reference amount (i.e., per 100 g), meaning that all other models (*n* = 23; 88%) used at least 2 different types of reference amounts in their algorithm (TABLE 4, TABLE 5, Supplemental Table 5). The most common combination, observed in 16 models (62%), was the use of the per 100 g reference amount along with the per 100 ml reference amount, depending on whether products are in the solid or liquid form (Supplemental Table 5).

#### Information on the validation of NP models

Validity testing of NP models can be conducted in different ways, which include, but are not restricted to, content, face, and criterion-related validity [[58], [59], [60]]. It was not possible to identify information on any form of validity testing for 58% of the included NP models (*n* = 15; Table 6). Of these, 5 models were, however, considered as having been “indirectly” validated because they represented adaptations from at least one other existing model that had previously been validated. Within the 11 models (42%) for which some degree of validity testing has been identified, 10 models were classified as having “limited” validity, meaning that information was found on only 1 or 2 forms of less robust validity testing (e.g., face validity with consumers and/or content validity with experts), regardless of whether the model may or may not have been based on other models. In fact, 8 of these 10 models (80%) with “limited” validity were based on at least one other NP model. Content validity was the most frequent form reported, and it had primarily been conducted by applying a model to a nationally generated list of food products (e.g., 100–200 foods frequently marketed to children) to be analyzed and commented on by food or nutrition experts in order to identify potential practical problems and/or by holding technical meetings with experts to discuss the models’ components (i.e., science content behind the model). Only one model (Samoa Nutrient Profiling System for Identifying Healthier and Less Healthy Food Options, no. 477) has also been assessed for construct validity. More specifically, agreement between this model and other well-known models was assessed to determine whether the model was appropriately restrictive. Of note, criterion-related validity, considered the most robust form of NP model validation, was not identified in any of the included models. Also, out of the 7 models primarily built for restricting food marketing to children*,* 86% (*n* = 6) were found to have at least a minimal degree of validity testing, compared with only 29% (*n* = 5) of the models primarily built for FOP food labeling (Table 4).

**TABLE 6:** Information on validity testing for the 26 included NP models^1^

Model number	Model name	Some degree of validity testing identified (Y/Limited/Indirect/N)^2^	Comments
Front-of-pack food labeling (*n* = 17)			
506	Brazil Warning Labels	N	Not identified
501	Brunei Healthier Choice Logo	N	Not identified
414	Canada Proposed Warning Labels	Limited	Limited (face validity)—In December 2016, Health Canada indicated that they had conducted focus groups to test some elements of their front-of-pack (FOP) proposal, namely the symbol design, size, and location, with 14 groups in 6 cities across Canada. The purpose was to assess how consumers understand and use the range of proposed nutrition symbols. In September 2017, Health Canada also hosted a 1-d meeting with stakeholders and experts to discuss FOP labeling evidence and options for the nutrition symbol design (https://gazette.gc.ca/rp-pr/p1/2018/2018-02-10/html/reg2-eng.html). These nutrition symbols are not shown in the proposed regulations; however, once selected, one nutrition symbol will be included in the final regulations that will be published in Canada Gazette, Part II, and will be inserted directly in the Food and Drug Regulations. The final symbol will be chosen based on the feedback from consultations and the outcomes of consumer research (https://www.canada.ca/en/health-canada/programs/consultation-front-of-package-nutrition-labelling-cgi/summary-of-proposed-amendments.html#shr-pg0).
496	Croatia Healthy Living Mark	N	Not identified
213	India Proposed Nutrient Profile Model	N	Not identified
503	Iran Traffic Light Labelling System	Indirect	Indirectly, since this model is based on the Traffic Light Labelling system from the United Kingdom (no. 41 in Labonté et al. [4]), which is validated.
485	Israel Green Label	Limited	Limited (content validity). A scientific committee (independent from the industry) was made up of leading nutrition and medical professionals from academia and the healthcare system and personnel of the Food Control Services and the Nutrition Division of the Ministry of Health to build the core principles and criteria behind the label. Several consultations were held, including consultations with international experts, and several meetings were also held, including one with representatives of the committee, public health experts from a medical association and academia, civil society organizations, and the food industry. Gillon-Keren et al. [22] indicates that "an evaluation plan is needed to assess the degree of acceptance of the positive front-of-pack labeling (FOPL) by the industry, retailers, and the public, and its impact on food consumption and on public health" (p. 10), suggesting that further validation of the model will likely be conducted in the future.
221	Israel Red Warning Labels	Limited	Limited (content validity) - According to Jones et al. [32], the government has led a consultation process that included input from independent food and nutrition experts and the food industry. Israel's Ministry of Health proposed to hold an evaluation with independent academics and also planned to obtain future sales data in order to assess the model's predictive validity.
511	Saudi Arabia Proposed Traffic Light Labelling System	Indirect	Indirectly, since this model is partly based on the Traffic Light Labelling system from the United Kingdom (no. 41 in Labonté et al. [4]), which is validated.
502	Malaysia Healthier Choice Logo	Indirect	Indirectly, since this model is based on several other models, some of which were found to be validated (Choices, no. 53, and WHO nutrient profile model for the Western Pacific Regional Office (WHO-WPRO), no. 334 in Labonté et al. [4]).
445	Mexican Warning Labels (Nutrisello)	N	Not identified
483	Peru Warning Labels	Limited	Limited (face validity) - A qualitative research study was apparently conducted through focus groups by the Ministry of Health with adolescents and parents in order to build the Manual on Advertising Warnings. However, details on this study were not found when searches were conducted on the Ministry website (https://www.gob.pe/minsa). Also, this model was based on other models, of which no. 388 from Labonté et al. [4] (PAHO nutrient profile model by the WHO Regional Office for the Americas) was found to be validated.
504	Sri Lanka Traffic Light Labelling System	N	Not identified
515	Thailand Healthier Choice Logo	N	Not identified
512	Tunisia Health Logo	Indirect	Indirectly, since this model is based on other models which were found to be validated (SAIN-LIM, no. 69 in Labonté et al. [4]) or indirectly validated (World Health Organization Nutrient Profile Model for the Eastern Mediterranean Regional Office, no. 333 in the current review).
484	Uruguay Warning Labels	N	Not identified
499	Zambia Good Food Logo	Limited	Limited (content validity) - The present model is an adapted version of a validated model (Choices, no. 53 in Labonté et al. [4]). Also, this model was developed following extensive collaboration with technical experts from the National Food and Nutrition Commission Zambia Bureau of Standards and Choices International. The nutrition criteria were based on international dietary guidelines from the World Health Organization and adapted to the Zambian nutrition context.
Restriction of marketing to children (*n* = 7)			
415	Canada Proposed Nutrient Profile Model for Marketing to Children	Limited	Limited (face validity) - Health Canada has conducted focus groups and consultations with stakeholders. However, the validation/review process was not completed yet as of June 2021. A final version of the proposed model and an implementation date are expected to be available, possibly in the upcoming year. Moreover, the draft model version 1.0 (2018 unpublished, received by the authors) was also applied to a Canadian food database in order to verify the relevance of the model in the actual Canadian market. The results highlighted the importance of introducing federal regulations for the restriction of marketing to children in Canada and the importance of including marketing on product packaging in the regulatory scope [47]. Also, this model was based on several other models which were found to be validated (Ofcom, no. 5; WHO Regional Office for Europe nutrient profile model (WHO-EURO), no. 335; PAHO nutrient profile model, no. 388 in Labonté et al. [4].)
481	Taiwan Recommendations on Sugar, Fat, Saturated fatty acids	N	Not identified
333	WHO Nutrient Profile Model for the Eastern Mediterranean Regional Office (WHO-EMRO)	Limited	Limited (content validity) - The present model is an adapted version of the validated model no. 335 (WHO Regional Office for Europe nutrient profile model (WHO-EURO)) included in Labonté et al. [4], and its regional adaptation consisted of a multi-step process. The first step was a field testing with 7 countries (Bahrain, Islamic Republic of Iran, Kuwait, Lebanon, Morocco, Oman, and Tunisia) by applying the model to a nationally generated list of 100–200 foods that are either frequently marketed to children or commonly consumed by children. Countries commented on various aspects, including food categories, nutrient thresholds, exclusions, and prohibitions. Adaptations included modifying and adding categories, nutrient thresholds, and sample/reference foods relevant for the Region. Though several adaptations were made, the draft model that was developed remained very similar to the European model.
336	WHO Nutrient Profile Model for the South-East Asia Regional Office (WHO-SEARO)	Limited	Limited (content validity) - The present model is an adapted version of the validated models no. 335 (WHO Regional Office for Europe nutrient profile model (WHO-EURO)) and no. 334 (WHO nutrient profile model for the Western Pacific Regional Office (WHO-WPRO)) included in Labonté et al. [4], and its regional adaptation consisted of a three-step process. The first step was a pilot testing of the draft model with 5 countries by applying the model to a nationally generated list of 100–200 foods that are either frequently marketed to children or commonly consumed by children. Countries commented on various aspects, including food categories, nutrient thresholds, exclusions, and prohibitions, and confirmed that foods categorized by the model are in line with national food-based dietary guidelines. The second step included a technical meeting with experts and country participants involved in the first step. The results of the field testing of the draft nutrient profile model were analyzed and reviewed. The recommendations and directions that were received from experts and country participants have been considered in the final model. The third step involved a consolidation of Member States’ comments, viewpoints, and specific requests to then review and finalize the model.
516	WHO Nutrient Profile Model for the African Region	Limited	Limited (content validity). The model is based on model no. 336 (WHO Nutrient Profile Model for the South-East Asia Regional Office (WHO-SEARO)) included in the current review, which is itself based on validated model no. 335 (WHO Regional Office for Europe nutrient profile model (WHO-EURO)) and no. 334 (WHO nutrient profile model for the Western Pacific Regional Office (WHO-WPRO)) included in Labonté et al. [4]. It has been pilot-tested in 9 countries with a list of 100 to 200 foods that are marketed to and/or commonly consumed by children in the country. Then, a regional consultation was held with experts from these 9 countries to review and finalize the model.
477	Samoa Nutrient Profiling System for Identifying Healthier and Less Healthy Food Options	Y	Validated in terms of content and convergent (construct) validity. Agreement between the current model and other well-known models (e.g., World Health Organization’s Nutrient Profile Model for the Western Pacific Region, no. 334 in Labonté et al. [4]) was assessed to determine whether the model was appropriately restrictive. Then, the proposed thresholds were tested in a food composition database to examine whether they targeted "discretionary foods." As described in Reeve et al. [52], consultations with experts and stakeholders also took place.
296	Sri Lanka Nutrient Profile Model	Limited	Limited (content validity) - The present model is an adapted version of model no. 336 (WHO Nutrient Profile Model for the South-East Asia Regional Office (WHO-SEARO)) included in the current review, which is itself based on validated model no. 335 (WHO Regional Office for Europe nutrient profile model (WHO-EURO)) and no. 334 (WHO nutrient profile model for the Western Pacific Regional Office (WHO-WPRO)) included in Labonté et al. [4]. This Sri Lankan adaptation [53] consisted of a multi-step process. According to the model's source reference (p. 6), its adaptation "involved a series of consultative meetings and workshops with content and context specialists and stakeholders from the health and non-health sectors, including the Ceylon Chamber of Commerce and other relevant food industries. The model was concluded considering the concerns and suggestions of all stakeholders."
Regulations of claims (e.g., health and/or nutrients) (*n* = 1)			
513	Tunisia Nutrient Content Claims	N	Not identified
Consumer education (*n* = 1)			
451	Slovenia Traffic Light System	Indirect	Indirectly, since this model is based on another model that was found to be validated (Traffic Light Labelling system, no. 41 in Labonté et al. [4]).

1 Abbreviations used: N, No; Y, Yes.

2 Definitions used for validity testing: YES means that information on multiple forms of validity testing was found for a given model, including at least one of the following more robust forms of validity testing: convergent or criterion-related validity. LIMITED indicates that information was found on only one or 2 forms of less robust validity testing for a given model (e.g., face validity with consumers and/or content validity with experts), regardless of whether the model may or may not have been based on other models. INDIRECT means that no specific information was found on the validity testing of a given model, but this model is, however, based on other model(s) that have previously been validated. NO indicates that information on the validation of a given model or of a model(s) on which this model is based could not be identified. Additional note: Different authors use either the term construct validity or the term convergent validity to describe similar forms of validation. Both terms are, therefore, used in combination in the current table.

## Conclusions

The overall aim of the current systematic review was to update a systematic review initially conducted by Labonté et al. in 2016 and published in 2018 [4], which aimed to retrieve NP models that have been developed or endorsed by authoritative bodies worldwide for use in government-led nutrition-related policies and regulations aimed at health promotion and NCD prevention.

Twenty-six new NP models have been retrieved from searches covering a 4-y period. The current review also shows that NP models have now been developed in countries and regions where no models were found in the previous systematic review (e.g., Iran Traffic Light Labeling System, no. 503, or Zambia Good Food Logo, no. 499). Such observations confirm that the field of nutrient profiling is in constant evolution worldwide and, therefore, reinforce the importance of having performed an update of the systematic review conducted by Labonté et al. [4] in 2016.

Interestingly, close to a quarter of the included models (*n* = 6, 23%; no. 213, 221, 296, 333, 336, and 515, the latter which replaced no. 302) originated from the reassessment of models that had previously been excluded from Labonté et al. [4] due to a lack of information available at that time. This suggests that building on previous work is highly relevant and crucial when conducting an update and that some models would have been missed if they had not been reassessed to verify whether information about them was now available.

The current review also shows an increase in the proportion of included NP models deriving from at least 1 other existing model compared with the previous systematic review [4] (i.e., 58% vs. 44%, respectively). This suggests that government bodies appear to increasingly put into practice the WHO’s recommendation to adopt or adapt existing models instead of building entirely new models, therefore limiting the possibility of inconsistencies between models, which can create confusion among users of NP models [61].

A lower variety of primary applications was identified for the NP models included in this review in comparison with the previous review (4 vs. 12, respectively). This might be explained, at least in part, by the fact that the current review comprised a lower absolute number of included models than the previous one (i.e., 26 vs. 78, respectively). It is also interesting to note that close to two-thirds (i.e., 65%) of models included in the current review were built for the same primary application, namely FOP food labeling. This proportion is much higher than the 15% of models (*n* = 12/78) built for FOP food labeling observed in the review by Labonté et al. [4], in which the most frequent primary application was school food (*n* = 27/78, 35%). This could be explained by the fact that the implementation of food labeling policies has become a key priority for several government bodies in recent years [5, 62, 63]. The second most predominant primary application in the current review, representing 27% of the included models, was the restriction of food and beverage marketing to children. Again, this proportion is twice as high as the one observed in Labonté et al. [4], at 13%. This suggests that protecting children from food marketing practices remains a critical topic for government bodies worldwide, particularly in recent years with the increase of food marketing in digital media [64, 65].

As previously observed by Labonté et al. [4], the number of food categories comprising nutrient criteria varied widely from one model to another, ranging from only one category (i.e., Food and Drink) in the Taiwan Recommendations on Sugar, Fat, and Saturated fatty acids (no. 481) to 84 categories in the Brunei Healthier Choice Logo (no. 501). FOP food labeling remained, as in Labonté et al. [4], the primary application for which the variation in the number of food categories was the highest. Although NP models with a high number of food categories might allow a better consideration of the varying nutritional composition of different food products, the implementation of such models might be more complex. More time and expertise are likely required to apply these models properly compared with the application of a single algorithm to any type of food. However, as mentioned by Sacks et al. [2], the use of several food categories is particularly helpful when the intent is to identify the “healthier” options within the same category (e.g., comparing coated vs. uncoated granola bars), which is frequently the aim of FOP food labeling, whereas a lower number of categories might be more relevant when a NP model is aimed at comparing different foods across a wide variety of products available to consumers, in the context of broader nutrition education.

As was also the case in Labonté et al. [4], the current review shows that all included NP models comprise nutrients to limit in their algorithm, with the top 3 nutrients to limit still being sodium, saturated fat, and total sugars. This is in line with dietary recommendations from many countries, which specifically consider these as the 3 main nutrients of public health concern [5, [66], [67], [68]]. However, although the systematic review by Labonté et al. [4] showed that 86% of the included models comprised nutrients or food components to encourage in their algorithm, less than a third (i.e., 31%) of models included in the current review comprised such nutrients. This could be explained by the most prominent primary application of NP models identified in the current review, namely FOP food labeling. Indeed, this type of nutrition-related policy frequently aims to restrict the consumption of less nutritious food products, as opposed to encouraging the consumption of more nutritious, nutrient-dense foods, which might be more typically put forward in policies about food offered in public settings such as schools. This is actually supported by the observation that the proportion of nutrient-specific systems typically targeting foods high in nutrients to limit (such as sugars, salt, or saturated fat), without taking beneficial nutrients into account, has highly increased in recent years within models built for FOP food labeling (i.e., 53% in the current review vs. 25% in the previous one). Similar to Labonté et al. [4], fiber and protein were within the top beneficial nutrients found in NP models that did include nutrients to encourage. However, the current review shows a shift toward calcium and iron within the top nutrients to encourage. Low dietary calcium intake is an identified risk factor for osteoporosis, which affects 1 in 3 women and 1 in 5 men over the age of 50 worldwide [69]. Given the aging of the population globally, calcium is likely becoming a micronutrient of increasingly higher public health concern. Regarding iron, its deficiency is one of the most common causes of anemia [70]. Worldwide, 42% of children less than 5 y of age and 40% of pregnant women are considered anemic [70]. This could potentially explain the interest in this micronutrient in new NP models. However, another possible explanation for the fact that calcium and iron were not necessarily included in previously identified models is that the inclusion of protein has often been used as a “proxy” for calcium and iron content, given its widespread presence in many jurisdictions’ nutrition facts table or nutrition information panel [71].

NP models are considered measurement tools and, therefore, need to be validated before being used in a specific target population [58, 59, 61, 72]. Again, similar to Labonté et al. [4], information on the validity of specific NP models could not be identified for over half of the models (i.e., 58%). Such observation does not necessarily mean that these models have never been validated. Given that the systematic review did not primarily focus on the validation of NP models, information on the validity testing of a model could be available in a publication that was not captured in our literature searches. Within models for which at least 1 form of validity testing has been identified, content validity conducted through expert consultations or through tests in a list of nationally representative foods analyzed by experts was the most common form reported, as opposed to construct (convergent) validity in the previous review by Labonté et al. [4]. This observation is somewhat surprising given that content validity represents a less robust form of validation than construct (convergent) validity [[58], [59], [60]]. This could be explained by the fact that in the current review, a higher proportion of new NP models was derived from other existing models that were already validated. Still, even if new NP models mostly represent adaptations of previously validated models, construct (convergent) validity testing, as well as criterion-related validity testing of such models, should be conducted in their target populations to support their use more efficiently.

The same limitations as those already described in the review by Labonté et al. [4] essentially apply to the current systematic review. These are nevertheless briefly summarized below. First, nutrient profiling is a rapidly growing and evolving field, and new NP models or adaptations to existing models can be published at almost any time. As previously mentioned by Labonté et al. [4], given the considerable time and resources required to identify NP models, assess their eligibility, and then extract and summarize their characteristics, keeping this systematic review entirely up to date is quite utopic. This review included 26 new NP models identified in a 4-y period ranging from 2016 to 2020; we can surely expect that other NP models have been developed since then.

Another limitation was that a single model could carry multiple names depending on the author or the publication in which it was found. To avoid missing some models, a precautionary approach was taken in which all models identified during the full-text assessment stage were first recorded separately into a list of potential models, even when some names were only very slightly different (e.g., 5-Colors vs. 5-CNL). Consultation of the source references of each potential model then allowed us to determine whether models with slightly different names were really different or simply the same model.

A third limitation is that the searches have been conducted in only one database of the grey literature in the current review, as opposed to 15 grey literature databases in the original review. This difference is primarily due to the fact that both reviews were not conducted in the same academic institution and that the institution in which the current review has been conducted does not provide access to many of the databases available in the other institution. To overcome this limit, the current review was, however, conducted in a higher number of databases of the peer-reviewed literature. Finally, the use of a low number of search terms in the literature searches might have contributed to the late identification of many additional potential models through online searches made during the eligibility assessment stage. This suggests that the search terms used might not have been precise enough to capture all of the possible applications of NP models, such as food labeling, food marketing, regulation of claims, school food, taxation, etc. The terms used in searches made in the peer-reviewed literature were nevertheless the same as in Labonté et al. [4], which ensures coherence between both reviews. Still, precising the search terms and, therefore, including search terms related to the most common applications of NP models would be highly relevant as part of eventual new updates.

In conclusion, this systematic review showed that the number of NP models developed or endorsed by authoritative bodies worldwide for applications in government-led nutrition-related policies aimed at health promotion and chronic disease prevention has kept growing throughout the years. Compared with the previous review conducted by Labonté et al. [4], there has been an increase in the number of NP models built in Asia, followed by the Americas, in addition to a shift toward NP models primarily built for FOP food labeling, followed by the restriction of food and beverage marketing to children. There has also been an increase in the proportion of new models that have a nutrient-specific rating system and that are adapted from at least one other existing model. As in Labonté et al. [4], all models include nutrients to limit in their algorithm, with sodium, saturated fat, and total sugars remaining the most frequently considered. The proportion of models that include nutrients or food components to encourage has, however, decreased substantially in recent years. Finally, over half of the included models had no information about their validity testing emerging from the retrieved publications. As NP models are increasingly used worldwide to support nutrition-related policies or regulations, this up-to-date resource proves to be unique and highly valuable for assisting users of NP models, such as researchers, health professionals, and policymakers, in the comparison and selection of models, so that these users can ultimately use models that are tailored to their needs. Given the high rate of proliferation of NP models, the creation of an online registry or database where groups could register their NP models should also be considered as a more practical future avenue.

## Author contributions

The authors’ contributions were as follows—VP and MEL designed research; CM, MT, JC, AL, and SP conducted research; CM, MT, and JC summarized results into the various tables and figures; CM wrote the paper’s first version; MT and MEL wrote the paper’s second version; MT, JC, AL, SP, VP, and MEL critically reviewed the manuscript; MEL has primary responsibility for final content. All authors have read and approved the final manuscript.

## Conflicts of interest

The authors report no conflicts of interest.

## Funding

Fonds de nutrition en santé publique de l’Université Laval (CM); INAF Starting funds for new investigators (MEL); Fonds de recherche du Québec – Santé (FRQS) Chercheurs-boursiers Junior 1 Salary Award (MEL; #266416); FRQS Young Investigator Establishment Grant (MEL; #280390); FRQS COVID supplement awarded to Chercheurs-boursiers Junior 1 recipients (MEL); Funds from the Food Quality Observatory/Ministère de la santé et des services sociaux du Québec (MEL; #GQ520912); Funds from the Faculté des sciences de l’agriculture et de l’alimentation - Soutien facultaire - Jeune Chercheur (MEL; #DC120930). Funders had no role in the study design, in the collection, analysis, and interpretation of data, in the writing of the manuscript, or decision to submit the manuscript for publication.

## Data availability

Data described in the manuscript will be made available upon request to the corresponding author.

## Appendix A Supplementary data

Multimedia component1 Multimedia component1

Multimedia component2 Multimedia component2

Multimedia component3 Multimedia component3
