# Supplementary material for: Systematic Review of Nutrient Profile Models Developed for Nutrition-Related Policies and Regulations Aimed at Noncommunicable Disease Prevention —An Update
Source: Adv Nutr. 2023 Aug 31;14(6):1499–522. doi: 10.1016/j.advnut.2023.08.013 (PMC10721541; doi:10.1016/j.advnut.2023.08.013)
Supplement: Multimedia component1 [file mmc1.pdf]

SUPPLEMENTAL TABLE 1 Details on search terms used, filters activated, and number of publications retrieved for each database as part of the peer-reviewed literature searches conducted on December 02, 2018 and September 02, 2020[1].

Peer-reviewed literature databases	Search terms specifically used	Filters activated	Number of publications retrieved
Pubmed	nutrient profil* OR nutritional profil* OR nutrition profil*	Publication date: from 2016/05/26 to 2020/09/02; Search field: Title / Abstract; Human	355
Embase[2]	(nutrient profil*) OR (nutritional profil*) OR (nutrition profil*)	Publication date: from 2016/05/26 to 2020/09/02 Search field: Title / Abstract; Human	529
Medline[3]	"nutrient profil*" OR "nutritional profil*" OR "nutrition profil*"	Publication date: from 2016/05/26 to 2020/09/02 Search field: Title / Abstract; Human	204
Cochrane	“nutrient profil*” OR “nutritional profil*” OR “nutrition profil*”	Publication date: from 2016/05/26 to 2020/09/02 Search field: Title / Abstract	34
CINHAL	“nutrient profil*” OR “nutritional profil*” OR “nutrition profil*”	Publication date: from 2016/05/26 to 2020/09/02 Search field: Title / Abstract	235
Google Scholar	“nutrient profil*” OR “nutritional profil*” OR “nutrition profil*”	Publication date: from 2016 to 2020[4] Search field: Title / Abstract	19
Psyc-INFO	“nutrient profil*” OR “nutritional profil*” OR “nutrition profil*”	Publication date: from 2016/05/26 to 2020/09/02 Search field: Title / Abstract; Human	222
**Total**			1598

[1] All electronic databases were accessed from the links provided on the Université Laval Library’s main web page (https://www5.bibl.ulaval.ca/).
[2] The search terms used for this database did not include the “adj” filter as in the previous systematic review by Labonté et al. 2018 (Labonté ME, Poon T, Gladanac B, et al. Nutrient Profile Models with Applications in Government-Led Nutrition Policies Aimed at Health Promotion and Noncommunicable Disease Prevention: A Systematic Review. Adv Nutr 2018;9(6):741-88. doi: 10.1093/advances/nmy045). After several tests, the “adj” filter has been removed in order to obtain more search results and avoid missing relevant publications.
[3] Medline accessed via Ebsco.
[4] In this database, it was not possible to choose the starting month of the inquiry. Therefore, duplicates for the first 6 months of year 2016 were removed manually.
