# Supplementary material for: Systematic Review of Nutrient Profile Models Developed for Nutrition-Related Policies and Regulations Aimed at Noncommunicable Disease Prevention —An Update
Source: Adv Nutr. 2023 Aug 31;14(6):1499–522. doi: 10.1016/j.advnut.2023.08.013 (PMC10721541; doi:10.1016/j.advnut.2023.08.013)
Supplement: Multimedia component2 [file mmc2.pdf]

SUPPLEMENTAL TABLE 2 Details on the search strategy used, filters activated, and number of publications retrieved as part of the grey literature search conducted on December 02, 2018 and September 02, 2020[1].

**Grey literature database**	**Search terms specifically used**	**Filters activated**	**Number of publications retrieved**
Web of Science[2]	"nutrient profil*" OR "nutritional profil*" OR "nutrition profil*"	Publication date: from 2016/05/26 to 2020/09/02 Search field: Title / Abstract; Human. Web of Science categories: (nutrition dietetics OR agricultural economics policy OR public environmental occupational health OR food science technology OR agriculture multidisciplinary OR education educational research OR behavioral sciences)	936
**Total**			936

[1] This electronic database was accessed from the links provided on the Université Laval Library's main web page (https://www5.bibl.ulaval.ca/).

[2] The search in this database did not include the more precise filters previously used in the systematic review by Labonté et al. 2018 (Labonté ME, Poon T, Gladanac B, et al. Nutrient Profile Models with Applications in Government-Led Nutrition Policies Aimed at Health Promotion and Noncommunicable Disease Prevention: A Systematic Review. Adv Nutr 2018;9(6):741-88. doi: 10.1093/advances/nmy045). In the current review, many of the filters have been removed because Web of Science was the only grey literature database consulted, and the research team wanted to get broad results as opposed to results related to conference abstracts only.
